# Secondary Infection of Adverse Local Tissue Reaction Leading to Amputation in a Patient With a Modular Knee Endoprosthesis

**DOI:** 10.5435/JAAOSGlobal-D-24-00293

**Published:** 2025-03-14

**Authors:** Mikaela H. Sullivan, Paul J. Gagnet, Joshua R. Labott, Diva R. Salomao, Matthew T. Houdek

**Affiliations:** From the Department of Orthopedic Surgery (Dr. Sullivan, Dr. Gagnet, Dr. Labott, Dr. Houdek); and the Department of Laboratory Medicine and Pathology (Dr. Salomao), Mayo Clinic, Rochester, MN.

## Abstract

Three years after endoprosthetic reconstruction of the proximal tibia, a patient presented with a skin ulceration near the surgical site. The knee was not clinically infected, but the patient had elevated serum cobalt levels. Dermatological evaluation diagnosed a friction ulcer. The ulcer failed to heal, and the patient underwent débridement and local flap advancement. Cultures grew *Staphylococcus epidermidis*, and they were treated with antibiotics. The ulceration recurred and repeated limb salvage was discussed, although eventually, the patient elected to undergo amputation. Pathology from the resected ulcer showed fibrinoid necrosis with aseptic lymphocytic vasculitis–associated lesion.

Adverse local tissue reaction (ALTR) to metal debris is often described as a combination of tissue necrosis and aseptic lymphocytic vasculitis–associated lesion (ALVAL). This is often seen in the setting of fretting and mechanically assisted crevice corrosion of modular junctions in patients with cobalt-chromium arthroplasties or in the setting of metal-on-metal arthroplasties.^1-3^ In patients with hip implants at risk of ALTR, 60% can have muscle necrosis observed at the time of revision surgery.^4^ ALVAL was first described in 2005 as a concept of a lymphocyte-dominated immune response in the periprosthetic soft tissues secondary to metal wear debris or corrosion from arthroplasty implants containing cobalt (Co) and chromium (Cr), subsequently leading to tissue necrosis.^5-7^

Owing to their off-shelf availability, relative ease of use, durability, and modularity, massive endoprostheses are commonly used for limb salvage after oncologic resections and in revision total joint arthroplasty.^8-12^ These implant systems are commonly made of Co, Cr, and titanium alloys, ^13-15^ and previous studies have shown that patients with these implant systems can have elevated serum and whole blood concentrations of Co and Cr.^16-20^ ALTR has previously been shown to develop in patients with a distal femoral endoprosthesis, leading to a pseudotumor and tissue destruction; however, these patients had a metal-on-metal hinges.^17^ We report a case of a patient who developed an ulceration over their proximal tibia after endoprosthetic reconstruction of the proximal tibia, which ultimately failed repeat limb salvage attempts. At the time of amputation, the ulceration and surrounding soft tissues were found to be involved by ALTR.

The patient was made aware that their case was being submitted as a case report, and they gave their consent to this.

## Case Report

A 33-year-old woman returned to our clinic 3 years after a proximal tibia endoprosthetic reconstruction (Global Modular Replacement System, Stryker Orthopaedics), which was performed for a pathologic fracture in the setting of a giant cell tumor of bone with a secondary aneurysmal bone cyst. At the time of surgery, the initial surgery, the patient's body mass index was 29.47 kg/m^2^. During surgery, the patient had a medial gastrocnemius flap; however, the skin was closed primarily over the wound. She had done well postoperatively and returned to her normal activities with full knee extension and pain-free weight bearing.

**Figure 1:**
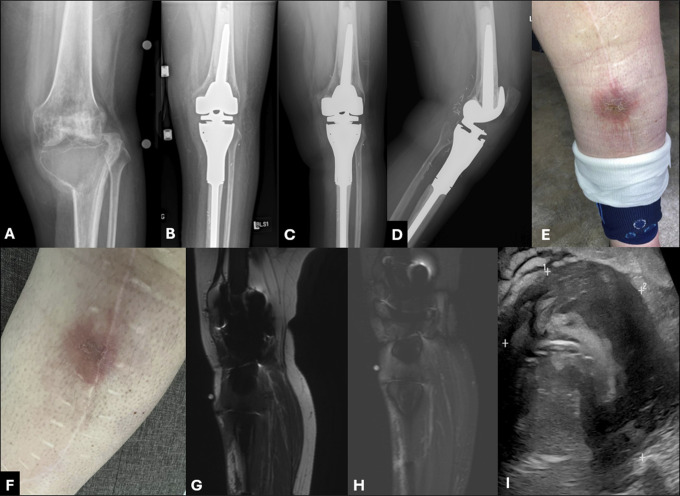
Preoperative AP radiographs and images showing a pathologic fracture of the proximal tibia through a large lytic lesion (**A**) with pathology showing giant cell tumor of bone. The patient was treated with a cemented proximal tibia endoprosthesis (**B**) and was doing well for 3 years. At 3 years postoperatively, the patient called in with concerns of their surgical incision. Radiographs were repeated (**C** and **D**) and showed no signs of implant loosening. Laboratory studies were negative for an acute infection; however, the patient developed a wound over the incision (**E**). They were treated empirically with oral antibiotics locally for a concern for a cellulitis. The erythema decreased; however, the wound persisted (**F**). Metal-suppression MRI with and without contrast was performed and on sagittal T1 (**G**) and STIR (**H**) images showed no signs of an abscess. However, owing to the limitations of metal-suppression MRI, an ultrasonography was recommended. An ultrasonography showed no fluid collection to suggest an abscess; however, there was a hypoechoic mass (**I**).

The patient began to have some discomfort in the anterior knee after localized trauma and noticed an area of erythema (Figure 1) 3 years postoperatively. She was evaluated in a local emergency department, with concern for cellulitis, and she was placed on an oral antibiotic. The oral antibiotic failed to resolve her erythema and discomfort, and the area of erythema progressed with expansion and drainage from the skin. During this time, knee range of motion was pain free. Owing to concern for a mass, an MRI scan was obtained with and without contrast, with metal suppression, and an ultrasonography was performed locally to evaluate the area for an abscess which was negative. Owing to persistent wound, she was evaluated by her local Dermatology team. The wound was swabbed and grew *Staphylococcus epidermidis* (*S. epi*), a diagnosis of cellulitis was provided, and she was placed on oral antibiotics.

The patient was evaluated in our orthopaedic clinic 10 days after taking any antibiotics and was seen by Orthopedic Infectious Disease. At that time, her inflammatory markers were mildly elevated (erythrocyte sedimentation rate 25 mm/h, C-reactive protein 7.8 mg/L) with pain-free range of motion of the knee without an effusion. Serum Co was 11.4 ppb, and Cr was <1 ppb. The ulceration was still present, and an aspiration of the knee was discussed. Shared decision was made for continued observation and follow-up within 3 weeks off antibiotics. When she was re-evaluated by our teams, inflammatory markers had decreased (erythrocyte sedimentation rate 22 mm/h and C-reactive protein 5.6 mg/L) and range of motion was still pain free, yet she still had an ulceration. She was evaluated by our Dermatology team and given a diagnosis of a friction ulceration secondary to compression socks. The area was swabbed again and grew pan-sensitive *S. epi*. A repeat ultrasonography was obtained to look for a sinus tract, and a 5.8 × 4.7 cm solid-appearing mass was noted, without a sinus tract. The mass around the endoprosthesis was biopsied and showed fibroconnective tissue with necrosis; deep cultures from the mass were negative.

**Figure 2:**
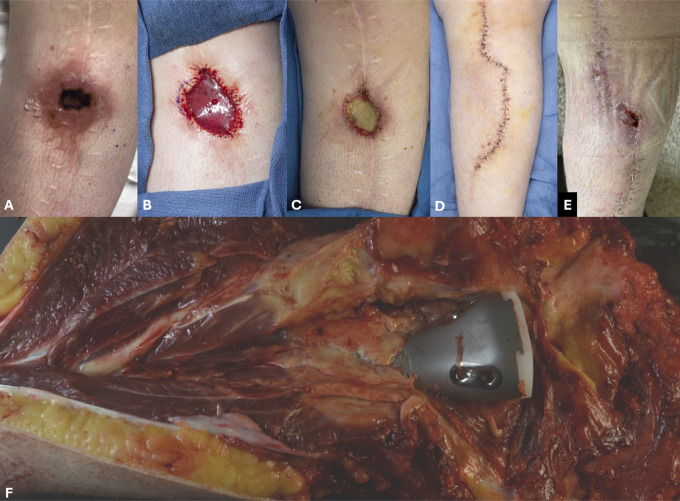
Images demonstrating the progression of ulceration (**A**). The determination was made to take the patient for débridement of the ulceration. At the time of surgery, the area appeared to have bleeding tissue and a dermal matrix was placed (**B**) along with a negative pressure dressing. At the time of dressing change 7 days later, the underlying tissue appeared to be necrotic (**C**) and the decision was made to proceed with a soleal rotation flap with primary closure (**D**). Most of the wound healed; however, in the area of the previous ulceration, another ulcer developed (**E**). At this point, additional revision was discussed with the patient and they elected to proceed with an above-knee amputation. At the time of amputation, a thick layer of necrotic tissue was noted around the implant surface.

**Figure 3:**
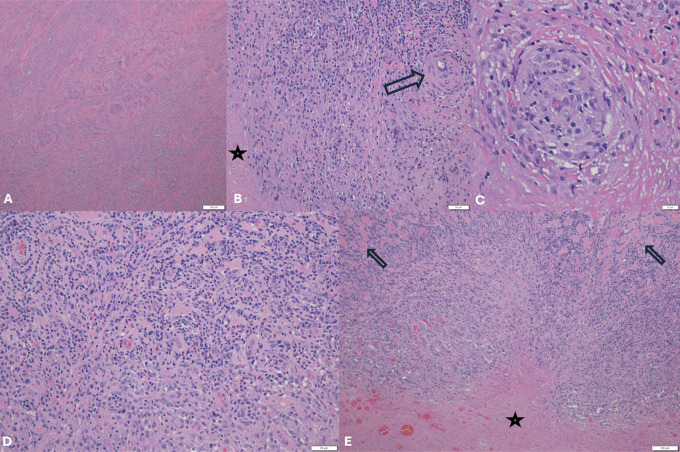
Images of hematoxylin and eosin (HE)–stained tissue samples showing extensive necrosis in the tissue obtained at the time of initial débridement of the ulceration (**A**—×100), reported as fat necrosis. At the time of amputation, tissue obtained from the ulceration and around the implant surface (**B**—HE; ×100) showed necrosis (star) and lymphohistiocytic infiltrate with intermixed eosinophils and a vessel showing narrowing of the lumen (arrow). High-power view shows blood vessel with concentric appearance secondary to lymphohistiocytic infiltrate causing thickening of the wall (**C**, HE ×400). Detailed view of lymphohistiocytic infiltrate displays vascular proliferation and intermixed eosinophils consistent with ALVAL (**D**, HE ×200). Low-power view reveals extensive necrosis (star) and lymphohistiocytic infiltrate involving skeletal muscle (arrows) and adjacent soft tissue (**E**, HE ×100) consistent with ALVAL. ALVAL = aseptic lymphocytic vasculitis–associated lesion.

The wound failed to heal with conservative wound care, and the decision was made to proceed with staged irrigation and débridement with placement of a dermal substitute (Figure 2). Cultures from the first-stage débridement of the ulceration grew *S. epi* and *Corynebacterium aurimucosum/minutissimum.* Both bacteria were susceptible to doxycycline, and they were treated with this for 2 weeks. The dermal substitute failed to incorporate, and the patient underwent a soleus advancement flap with primary wound closure. At the time of each procedure, tissue was débrided and a diagnosis of fat necrosis was given. Intraoperative frozen sections were obtained during each procedure, and all were negative for acute inflammation. During the local flap coverage, the thick area of tissue was débrided and the implant was not exposed. Cultures continued to grow *S. epi*., which was susceptible to doxycycline and she was placed on an extended course of antibiotics. The wound of the local flap failed to heal over the area of previous ulceration. Repeat limb salvage attempts were discussed with the patient to undergo a free flap coverage, two-stage vs. single-stage exchange with flap coverage, and rotationplasty versus a transfemoral amputation. The patient elected for rotationplasty but knew that an amputation was possible. At the time of surgery, a thick area of necrotic tissue was noted surrounding the endoprosthesis and popliteus vessels. Attempt was made to free the vessels from the necrotic tissue; however, this was not possible, and the patient underwent a transfemoral amputation. Pathology from this tissue demonstrated necrotic tissue most consistent with ALVAL (Figure 3). The wound healed uneventfully, and the patient now uses a prosthesis.

## Discussion

ALTRs are known complications of metal-on-metal and modular arthroplasty reconstructions. Tissue necrosis has historically focused on the soft tissues surrounding the hip in the form of pseudotumors. This report shows that ALTR can lead to skin and soft-tissue necrosis, which could affect the ability for wound healing overlying a proximal tibia endoprosthesis.

Amputation after limb salvage surgery most commonly occurs in patients with a proximal tibia endoprosthesis.^21,22^ Although local recurrence is the most common indication for amputation at other anatomic sites, local recurrence and infection have a similar incidence of amputation in the proximal tibia.^21^ The risk of infection and amputation for patients with a proximal tibia arthroplasty has been reduced with the utilization of a gastrocnemius flap; however, even with the additional soft-tissue coverage, amputations still occur and infection remains the most common cause of failure.^8,21,23,24^

Pseudotumors and soft-tissue necrosis have previously been reported around revision total knee arthroplasties secondary to catastrophic wear of the polyethylene or malpositioning.^25,26^ In addition, these fluid collections have been discovered incidentally around an endoprosthesis.^27^ Although we did not conduct retrieval analysis on the implant from this patient, the Global Modular Replacement System implant has been noted to have fretting and corrosion at the modular interface retrieval analysis,^28^ making a case for mechanically assisted crevice corrosion leading to ALTR in this patient.

The soft-tissue destruction associated with ALTR increases the risk of prosthetic joint infection (PJI), and often PJI and ALTR can have similar presentations.^29–32^ In addition, ALTR and the presence of particulate metal debris increase the risk of PJI.^29,31,33^ Owing to increasing evidence of ALTR occurring in patients with endoprosthesis, when a soft-tissue mass develops around an endoprosthesis, which is not a local recurrence, it could be thought of as an implant that is “at risk” for the development of a late infection similar to other metal-on-metal implants. It is difficult to know whether the ulceration was due to the infection or ALTR; however, its metal ions are cytotoxic to stem cells but not *S. epi*,^32^ which could impair the body's ability to heal a wound in this situation.

This case shows that ALTR should be considered on the differential diagnosis for patients presenting with a mass and tissue necrosis around an endoprosthesis, and that this process is not limited to total hip arthroplasties. In this patient, additional limb salvage was attempted, but failed secondary to the underlying ALVAL. Patients should be cautioned on the risk of amputation when cutaneous manifestations of this process occur, and patients with elevated serum metal ion levels warrant observation for the development of this condition.
